# Neutron imaging to study the influence of flow fields and porous electrodes on concentration distributions in redox flow cells

**DOI:** 10.1039/d5se00844a

**Published:** 2025-08-05

**Authors:** Maxime van der Heijden, Rémy Richard Jacquemond, Emre Burak Boz, Pierre Boillat, Antoni Forner-Cuenca

**Affiliations:** a Electrochemical Materials and Systems, Department of Chemical Engineering and Chemistry, Eindhoven University of Technology P.O. Box 513 5600 MB Eindhoven The Netherlands r.r.jacquemond@tue.nl e.b.boz@tue.nl a.forner.cuenca@tue.nl; b Department of Chemical Engineering, University of Waterloo Waterloo ON Canada maxime.vanderheijden@uwaterloo.ca; c Eindhoven Institute for Renewable Energy Systems, Eindhoven University of Technology P.O. Box 513 5600 MB Eindhoven The Netherlands; d Electrochemistry Laboratory, Paul Scherrer Institut Forschungsstrasse 111, Villigen PSI CH-5232 Switzerland pierre.boillat@psi.ch; e Laboratory for Neutron Scattering and Imaging, Paul Scherrer Institut Forschungsstrasse 111, Villigen PSI CH-5232 Switzerland

## Abstract

Understanding reactive mass transport in redox flow reactors is key to improving performance, yet conventional characterization techniques often rely on cell-averaged metrics and fail to resolve local transport phenomena. In this study, we employ *operando* neutron radiography to visualize concentration distributions in redox flow cells with non-aqueous electrolytes, leveraging the high attenuation of hydrogen-containing organic molecules and boron-containing supporting ions. Symmetric flow cell experiments were conducted with three electrode types (paper, cloth, and a hierarchical porous electrode fabricated by non-solvent induced phase separation), and two flow field designs (parallel and interdigitated). We find that for kinetically facile electrolytes with low ionic conductivity and with parallel flow fields, electrodes with large pores in the though-plane direction (*i.e.*, carbon cloth) augment the current output. Additionally, interdigitated flow fields sustain higher currents than parallel flow fields at a fixed potential and flow rate due to enhanced convective transport. Despite significant differences in macroscopic performance among the studied materials, the concentration profiles within the cell showed only minor variations within the studied operating conditions and imaging configuration. The cloth electrode and interdigitated flow field exhibited slightly more uniform concentration profiles across the electrode thickness compared to the paper electrode with the parallel flow field. In contrast, the phase-separation electrode displayed more steep concentration profiles and a stronger dependency on polarity reversal. Neutron radiography further uncovered critical secondary effects, including salt precipitation and flow field underutilization. These findings highlight the potential of *operando* imaging to inform the design and operation of electrochemical reactors for a range of technologies.

## Abstract 2

This study uses *operando* neutron imaging and electrochemical data to reveal how flow field geometry and electrode structure shape concentration profiles and transport dynamics in organic redox flow cells.

## Graphical abstract

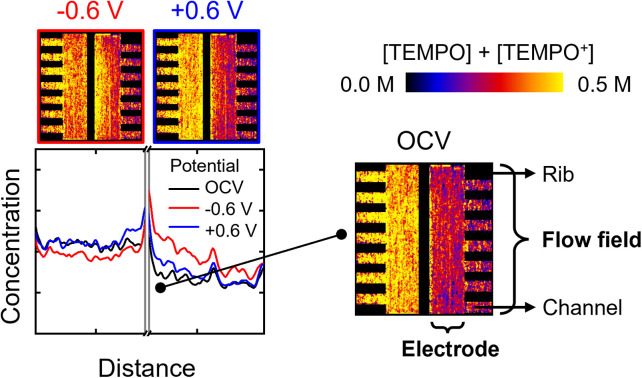

## 1 Introduction

Redox flow batteries (RFBs) are rechargeable electrochemical systems that are promising for large-scale stationary energy storage due to their design flexibility which enables independent scaling of power and energy, simple manufacturing, and system adaptability (*i.e.*, different electrolytes and operating conditions).^1–5^ In RFBs, electrolytes containing dissolved or suspended redox active species are stored in external tanks and pumped through the electrochemical reactor where the active species undergo redox reactions to charge and discharge the battery. The electrochemical stack comprises current collectors with flow fields, porous electrodes, and membranes, which together determine the power output of the system. Among various technical challenges (*e.g.*, performance, lifetime, power losses (overpotentials)) in the battery limit the system efficiency and lead to increased capital costs.^6^ Enhancing power density in electrochemical cells requires optimizing reactor design, material selection, and operating conditions to improve reactive mass transport.^5^ The porous electrodes control the electrolyte distribution, provide active surfaces for reactions, cushion mechanical compression, and facilitate mass, charge, and heat transport,^7,8^ whereas flow fields distribute the electrolyte to the porous electrodes, affecting pressure drop and electrode utilization.^8–11^ Understanding the interplay between mass transport and reactions is crucial for designing better reactors and materials. To this end, there is a growing interest in investigating the performance of conventional porous electrodes under specific reactor designs and conditions^3,10,12–19^ as the flow field design, electrolyte chemistry, and operating conditions significantly impact the electrode performance.^3,9^

Conventional techniques for evaluating electrochemical cell performance rely on electrochemical diagnostic tools, *e.g.*, polarization curves and electrochemical impedance spectroscopy, along with *ex situ* characterization methods such as scanning electron microscopy and X-ray photoelectron spectroscopy.^8^ However, local *operando* metrics, such as concentration distributions, are complex to resolve, which are essential for correlating material and reactor components to battery performance. Combining *operando* imaging diagnostics (*e.g.*, X-ray tomography,^20–23^ fluorescence imaging,^24^ (nuclear) magnetic resonance imaging,^25–28^ and neutron imaging^29–33^), with conventional electrochemical tools is a promising approach. Neutron imaging is particularly useful as it enables reactor-level, long-duration, and non-invasive imaging.^29^ As shown in our previous work,^30^ the relatively high attenuation of hydrogen and boron^34,35^ makes this technique suitable to study non-aqueous RFBs (NAqRFBs) with organic redox molecules. Due to the low attenuation of cell housing, neutron imaging enables the study of various reactor components by contrasting redox-active molecules, supporting ions, solvents, and gases, while requiring minimal cell modifications.^29,30^ However, the *operando* visualization of concentration distributions in microstructurally-diverse porous electrodes and flow fields remains underexplored.

**Fig. 1:**
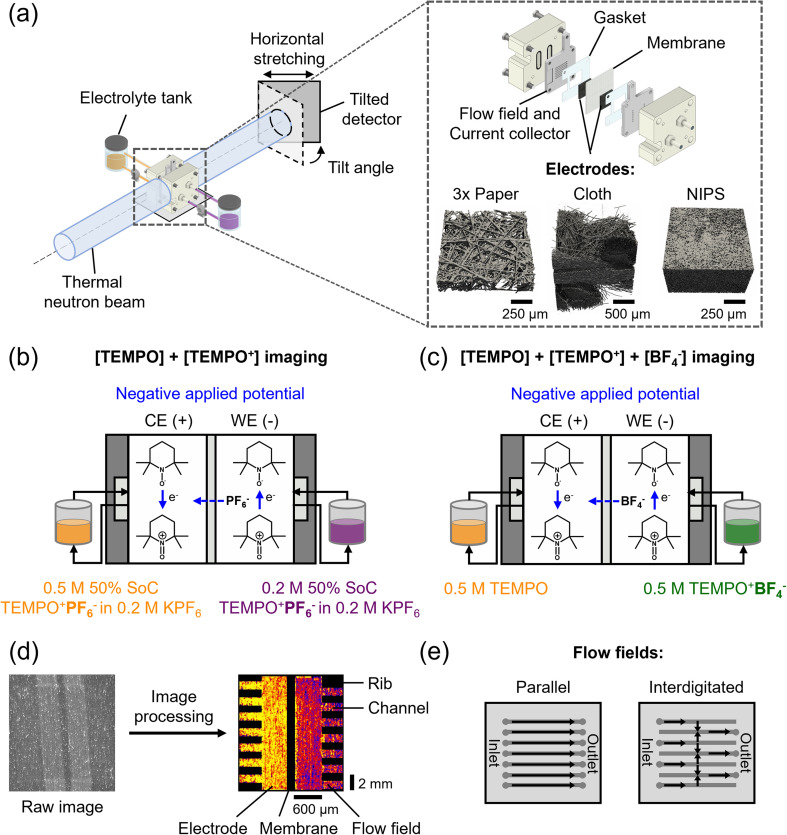
Schematic representations of (a) the neutron imaging set-up for cumulative concentration profiles of active species and supporting ions, together with the flow cell design, cell components, and the X-ray tomographic images of the electrode types: three stacked papers, a cloth, and an in-house manufactured NIPS electrode; (b and c) the non-aqueous cell design with chemical structures of the active species (TEMPO and TEMPO^+^), shown for a negative applied potential, and the different electrolyte tank compositions: (b) 0.5 M [TEMPO] + [TEMPO^+^PF_6_^−^] at 50% state-of-charge in 0.2 M KPF_6_ at the counter electrode (CE) and 0.2 M [TEMPO] + [TEMPO^+^PF_6_^−^] at 50% state-of-charge in 0.2 M KPF_6_ at the working electrode (WE), and (c) 0.5 M TEMPO at the CE and 0.5 M TEMPO^+^BF_4_^−^ at the WE; (d) simplified image processing steps from raw to processed image showing the flow fields, electrodes, and porous separator; (e) flow field types used: parallel and interdigitated.

In this work, we apply our previously developed method^30^ to resolve concentration profiles during flow cell operation, examining the influence of electrodes and flow fields on NAqRFB performance (Fig. 1a and e). By operating flow cells in the beamline, we correlate their macroscopic electrochemical performance with the resulting concentration profiles at the cell level. We investigate three porous electrodes with distinct microstructures: paper, cloth, and an in-house manufactured hierarchical porous electrode through non-solvent induced phase-separation (NIPS). Additionally, we study two flow field geometries, flow-by parallel (PFF) and interdigitated flow field (IDFF), to assess the influence of forced convection on the cell performance. Using two different electrolytes (Fig. 1b and c), we resolve the mass transport of redox molecules and supporting ions. We aim to illustrate the potential of neutron radiography to study the contributions of various reactor components to mass transport in *operando* flow cells by obtaining local concentration profiles.

## 2 Materials and methods

For more detailed descriptions of the experimental procedures used in this study, please refer to our previous work.^30^

### 2.1 Electrode structure and material properties

This study investigated two commercial carbon fiber-based porous electrodes (Table 1): the randomly organized Sigracet 39AA (Fuel Cell Store, three stacked electrodes of 280 μm each, compressed to 630 μm total thickness, 89% porosity,^3^ and 85% compressed porosity) and the highly ordered woven 1186 HCB Cloth (AvCarb, 1200 μm, compressed to 630 μm, 82% porosity,^13^ and 66% compressed porosity). Additionally, an in-house manufactured NIPS electrode (600 μm, compressed to 530 μm, 87% porosity,^36^ and 85% compressed porosity) was studied. NIPS electrodes are non-fibrous with interconnected pore networks, offering good performance in convection-driven flow cells. They offer diverse microstructures with a controllable pore size distribution (PSD), ranging from macrovoids to a porosity gradient, depending on manufacturing conditions, and are fabricated using a simple manufacturing process.^36,37^ A note about the discrepancy between the compressed electrode porosities can be found in Section S1 in the SI.

**Table 1:** Electrode properties for the paper, cloth, and NIPS electrodes

	SGL 39AA	AvCarb cloth	NIPS
Thickness [μm]	3 × 280	1200	600
Compressed thickness [μm]	630	630	530
Porosity [%]	89^3^	82^13^	87^36^
Compressed porosity [%]	85	66	85
Electrochemically active surface area [m^2^ g^−1^]	0.50^a^^3^	0.028^a^ (0.81^b^)^13^	2.9^a^^36^
Average pore size [μm]	63^3^	20, 100^13^	1–5^36^
In-plane permeability × 10^−11^ [m^2^]	7.4^3^	1.4^13^	1.0^36^
Forchheimer coefficient × 10^4^ [m^−1^]	3.2^3^	0.12^13^	7.7^36^

a Measured by electrochemical double-layer capacitance method.

b Measured by nitrogen sorption, using the Brunauer–Emmett–Teller method.

The electrodes were scanned using a laboratory micro-CT (Scanco Medical μCT 100 cabinet microCT scanner, holder type U50822 with a diameter of 9 mm and a height of 78 mm) at an isotropic resolution of 3.3 μm per voxel. The scans were conducted with a peak potential of 55 kVp, a current of 72 μA, 4 W, and a 0.1 mm aluminum filter. Between 433–1234 projection images were taken over 360°. The gray-scale images were processed with ImageJ using a two-dimensional median filter (radius of 2.0 pixels) and a K-means cluster segmentation filter to assign each voxel to the solid or void phase.

### 2.2 Electrolyte preparation

(2,2,6,6-Tetramethylpiperidin-1-yl)oxyl (TEMPO, Sigma Aldrich, 98%) was converted to the oxidized form 2,2,6,6-tetramethyl-1-piperidinyloxy-oxo (TEMPO^+^) by chemical oxidation with nitrosonium salts: nitrosonium hexafluorophosphate (NOPF_6_, Thermo Scientific, 95%) or nitrosonium tetrafluoroborate (NOBF_4_, Thermo Scientific, 98%), inside a nitrogen-filled glove box (MBraun, LABstar, O_2_ < 1 ppm, H_2_O < 1 ppm). 1.1 molar equivalents of the nitrosonium salt (15.42 g NOPF_6_ or 10.29 g NOBF_4_) were slowly added over 2 hours to TEMPO (12.52 g), all dissolved in acetonitrile (CH_3_CN, Sigma Aldrich, ≥99.9%), to prevent NO_*x*_ build-up.^3^ The acetonitrile was evaporated using a rotary evaporator (40 °C, gradual decrease from atmospheric pressure to vacuum) to obtain the TEMPO^+^PF_6_^−^ or TEMPO^+^BF_4_^−^ salts. Solutions of 20 mL were prepared for the two experimental configurations (Fig. 1b and c). For the experiments with the potassium hexafluorophosphate salt (KPF_6_, Thermo Scientific, 99%), 50% state-of-charge (SoC) solutions were prepared with different concentrations of TEMPO species (0.5 M and 0.2 M TEMPO/TEMPO^+^PF_6_^−^) in 0.2 M KPF_6_, dissolved in deuterated acetonitrile (CD_3_CN, Zeochem AG, 99.8%D). Deuterated acetonitrile was used to minimize the attenuation of the solvent on the overall transmission as deuterium has a 10 times lower cross-section compared to hydrogen (at 2200 m s^−1^ neutron velocity).^38^ For the experiments using the tetrafluoroborate (BF_4_^−^) supporting ions, solutions of 0.5 M TEMPO and 0.5 M TEMPO^+^BF_4_^−^ dissolved in CD_3_CN were prepared.

### 2.3 Neutron radiography

Neutron radiography experiments were conducted at the NEUTRA beamline in the Spallation Neutron Source facility of the Paul Scherrer Institute, Switzerland. The NEUTRA beamline operates with thermal neutrons emitted from a lead spallation target upon interaction with a proton beam (590 MeV energy, 1.5 mA proton current) and moderated by heavy water to reach thermal velocities (25 meV).^39^ The beamline was equipped with an in-plane imaging set-up with a tilted charge-coupled device camera detector (CCD, Andor, 30 s exposure time) to produce a stretched image in the horizontal transverse direction with respect to the incident beam (Fig. 1a), allowing higher spatial resolution across the flow field-electrode-separator domain.^40^ This resulted in a pixel size of 6 μm, translating to an effective resolution of 20 μm considering beam divergence and detector blurring. Attenuated neutrons were captured by a scintillator screen (10 μm thick, Gd_2_O_2_S : Tb), converted to visible light, and confined by the CCD detector. The imaging configuration allows us to resolve the concentration distribution locally through the electrode thickness (Fig. 1d) and provides deeper insights into coupled mass transport phenomena (convection, diffusion, migration) unattainable with conventional characterization methods.

### 2.4 Calibration experiments

*Ex situ* cuvette calibration measurements were performed to obtain the attenuation coefficients of various electrolyte solutions to quantify the total concentration in the electrochemical cells. The attenuation coefficients, *i.e.*, the attenuation of the neutron beam by the sample, were obtained with the Lambert–Beer law:^41^

1 *T* = e^−*σ*_*i*_*n*_*i*_*δ*^

where *T* is the transmitted intensity [−] corrected for the attenuation of an empty cuvette, *σ*_*i*_ the conventional microscopic cross-section [m^2^], *n*_*i*_ the number density of species *i* [m^−3^] which is a function of the concentration of species *i* (*C*_*i*_) [mol m^−3^] and Avogadro's number [mol^−1^] (*N*_A_, 6.02 × 10^23^ mol^−1^) by *C*_*i*_ = *n*_*i*_/*N*_A_, and *δ* the sample thickness [m] which was 1 cm for the cuvettes and *δ* = *L*_e_*ε*_e_ for the flow cell experiments, with *L*_e_ being the electrode width (17 mm) [m] and *ε*_e_ the electrode porosity at the applied compression [−]. The cuvette calibration measurements were performed to obtain the microscopic cross-sections for solutions of CD_3_CN (solvent reference), 0.2 M KPF_6_ (supporting electrolyte), and solutions of varying molarities (0.1, 0.2, 0.3, 0.4, and 0.5 M) of TEMPO, TEMPO^+^PF_6_^−^, and TEMPO^+^BF_4_^−^ to verify the linearity between the concentration and neutron attenuation, all dissolved in CD_3_CN. The calibration experiments were extensively discussed in our previous work,^30^ showing similar macroscopic cross-sections for TEMPO and TEMPO^+^PF_6_^−^ in CD_3_CN, KPF_6_ in CD_3_CN has similar neutron attenuation to CD_3_CN, and TEMPO^+^BF_4_^−^ has roughly double the macroscopic cross-section of TEMPO.

### 2.5 Flow cell configuration

The flow cell experiments were performed using a laboratory-scale system,^3,42,43^ with the cell mounted on a robotized platform 1–3 mm in front of the neutron detector. The cell was slightly modified (grooved flow fields and gaskets around the active area, see Fig. 1a) to enhance sensitivity by minimizing cell part attenuation without impacting electrolyte transport. Electrolyte solutions were pumped through the cell using a Masterflex L/S® peristaltic pump with an Easy-Load® II pump head and LS-14 tubing, connected to two 20 mL reservoirs. Custom-made flow cells featured machined polypropylene diffusers (McMaster-Carr) and graphite current collectors (G347B graphite, 3.18 mm thick, MWI, Inc.), milled with parallel (seven 16 × 1 × 0.5 mm^3^ channels parallel to the electrolyte flow) or interdigitated flow field designs (seven 16 × 1 × 0.5 mm^3^ channels parallel to the electrolyte flow, of which four inlet- and three outlet channels) (Fig. 1e). Electrodes (2.55 cm^2^ geometric area) were confined within incompressible polytetrafluorethylene gaskets (ERIKS, thicknesses of 210 μm and 110 μm were used). A Daramic 175 porous separator (SLI Flatsheet Membrane, 175 μm) was used to separate the half-cells and the cell was tightened to 2 Nm with a torque-controlled wrench.

To compare the flow cell performance between the different electrodes, the electrolyte flow rate was controlled. Evaluated flow rates were 15.3 and 5.1 mL min^−1^ for the paper electrode, 15.3, 5.1, 45.9, and 1.7 mL min^−1^ for the cloth electrode, and 12.8 and 4.5 mL min^−1^ for the NIPS electrode, performed in descending order to remove residual gas bubbles and enhance electrode wetting. Furthermore, two flow field designs, PFF and IDFF, were investigated with the paper electrode at constant flow rates (15.3 and 5.1 mL min^−1^). The electrolyte velocity can be obtained with eqn (2) for the PFF and eqn (3) for the IDFF, shown for both the electrolyte velocity in the channel and in the electrode. However, calculating the velocity through the electrode with a PFF is challenging, and often a negligible electrolyte velocity is assumed.^12,15^2
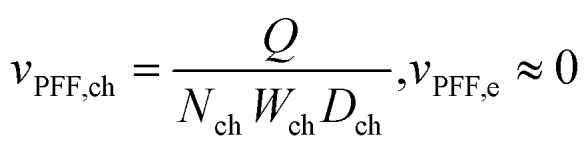
3
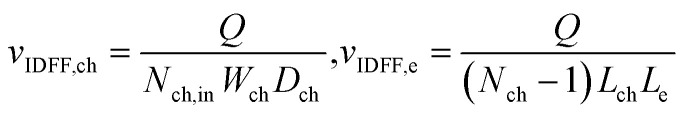
with *v* being the electrolyte velocity [m s^−1^] with the PFF or IDFF in the flow channel or the electrode, *Q* the electrolyte flow rate [m^3^ s^−1^], *N*_ch_ the total number of channels in the flow field configuration (7 channels) [−], *N*_ch,in_ the number of inlet channels (4 channels for the IDFF) [−], *W*_ch_ the channel width (1 × 10^−3^ m) [m], *D*_ch_ the channel depth (5 × 10^−4^ m) [m], *L*_ch_ the channel length (1.6 × 10^−2^ m) [m], and *L*_e_ the compressed electrode thickness (6.3 × 10^−4^ m or 5.3 × 10^−4^ m) [m].

### 2.6 Electrochemical performance

For the electrochemical cells, the procedure started with measuring the open circuit voltage (OCV) to monitor species diffusion between the half-cells for 20 min for the KPF_6_ supporting salt experiments or for 1 hour for the BF_4_^−^ supporting ion experiments. OCV experiments were performed at 15.1 mL min^−1^, except for the NIPS electrode (12.8 mL min^−1^). The electrochemical sequence involved applying −0.3 V, +0.3 V, −0.6 V, and +0.6 V using a BioLogic VSP-3e potentiostat, with each step lasting 20 min at each flow rate (Section 2.5), resulting in an electrochemical protocol of 180–220 min for the paper electrode for both flow fields (Fig. S6 and S9). The NIPS electrode followed the same protocol (Fig. S1c), but due to a beam shutdown, some images were missing (−0.3 V and +0.3 V at 4.5 mL min^−1^ for the BF_4_^−^ experiment at ∼90 min, the −0.6 V and +0.6 V sequences at both flow rates were repeated, Fig. S3c). There was also a beam shutdown for the IDFF, resulting in a time jump of 40 min (KPF_6_ experiment at 100–140 min, Fig. S6b). The cloth electrode (Fig. S1b and S3b) was investigated at two additional flow rates (at −0.6 V and +0.6 V for 20 min), resulting in a sequence of 260–300 min. Neutron radiographs were collected throughout. Both supporting salt (KPF_6_) and supporting ion (BF_4_^−^) experiments were performed on the same cell, with a rinsing step in between using 0.5 M TEMPO in CD_3_CN, except for the paper–PFF configuration.

### 2.7 Image processing

The image processing steps to obtain the final concentration distribution color maps (Fig. 1d) were performed using a Jupyter Notebook script. Transmission data was corrected for detector background effects, beam variations, changes in the sample position, interactions of the beam with other cell components, and camera noise, in a subtractive manner. Five image sets were obtained: raw images, black body, open beam, dark current, and reference images. The raw images of the cell with the electrolyte solution (low attenuating salt KPF_6_ or highly attenuating supporting ion BF_4_^−^) were processed as follows:

(1) *Dark current correction*: eliminates electronic bias within the camera circuity.

(2) *Filtering*: a white spot filter corrects gamma radiation effects and a Gaussian filter reduces statistical noise.

(3) *Open beam correction*: corrects spatial variations in beam intensity.

(4) *Registration*: accounts for cell movements due to relaxation or thermal dilation.

(5) *Intensity correction*: adjusts for beam intensity variations.

(6) *Scattered background correction*: corrects for neutrons scattered by the detector and cell.

(7) *Referencing*: obtains the transmission values of the species of interest.

(8) *Image rotation*: aligns the cells horizontally.

(9) *Cell mask application*: shows the region of interest (flow fields and electrodes, Fig. 1d). A mask is applied over the porous separator because the active species concentration cannot be accurately determined due to the separator's low thickness and high neutron attenuation.

(10) *Concentration calculation*: uses Lambert–Beer law (eqn (1)) to translate neutron transmission to concentration. With a “concentration factor” (−*σ*_*i*_*n*_*i*_*δ*) of −0.891 L mol^−1^ for the paper and NIPS electrodes, and −0.686 L mol^−1^ for the cloth electrode (lower porosity).

An extensive description of the image processing steps can be found in our previous work.^30^

## 3 Results and discussion

### 3.1 Influence of the electrode structure

#### 3.1.1. Transport of the active species

**Fig. 2:**
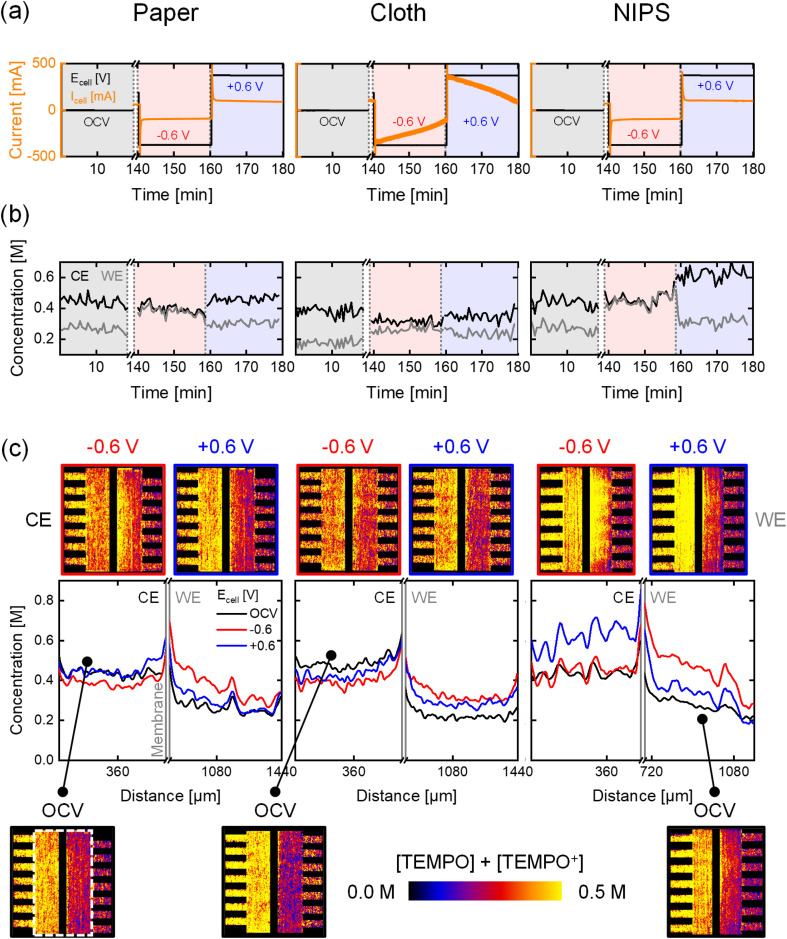
*Operando* neutron imaging of the active species transport with the low attenuating KPF_6_ supporting salt for three electrode types: paper, cloth, and an in-house manufactured NIPS electrode, at an inlet flow rate of 5 mL min^−1^ and evaluated at OCV, −0.6 V, and +0.6 V. (a) The potential applied and current output of the electrochemical cells. (b) The averaged concentration profiles over time in the counter electrode (CE) and the working electrode (WE). (c) The cumulative active species (TEMPO and TEMPO^+^) concentration profiles over the electrode thickness with the averaged snapshots of the cell after image processing, with on the counter electrode 0.5 M [TEMPO] + [TEMPO^+^PF_6_^−^] at 50% state-of-charge in 0.2 M KPF_6_ and at the working electrode 0.2 M [TEMPO] + [TEMPO^+^PF_6_^−^] at 50% state-of-charge in 0.2 M KPF_6_. The white dashed box in the OCV image for the paper electrode shows the area of the images that are plotted in the concentration profiles, *i.e.*, the porous electrode, excluding the flow field.

As described in our previous work,^30^ imaging of the active species TEMPO and TEMPO^+^ can be achieved using the low attenuating supporting salt KPF_6_. The movement of active species due to concentration gradients and voltage bias can be visualized by tracking their concentration distribution through the reactor owing to the large neutron attenuation of hydrogen-containing TEMPO species. Accordingly, electrolyte tanks with 50% SoC TEMPO and TEMPO^+^ with 0.5 M on the counter electrode (CE) side and 0.2 M on the working electrode (WE) side were used, both with 0.2 M KPF_6_ for ionic conductivity and minimal neutron attenuation (Fig. 1b). Fig. 2 shows electrochemical data and concentration profiles over the electrode thickness from the CE to WE for the three electrode types at OCV, −0.6 V, and +0.6 V at 5 mL min^−1^. The images and profiles were averaged over 20 min for each applied potential and represent a pixel average (over the electrode length and width) in the direction of the neutron beam. Higher flow rate and lower potential profiles are shown in Section S1.1. In generating the concentration distribution profiles (Fig. 2c), only the porous electrode region was considered in the averaging process. Flow fields, including rib areas (porosity = 0) and flow channels (porosity = 1), were excluded to avoid skewing the results. The flow field domain typically exhibits lower average neutron attenuation which dilutes the concentration signal. While visually retained in the images for contextual awareness, they do not contribute to the plotted averages. Fig. 2c includes a reference box to indicate the specific region analyzed in the 1D concentration plots, which was applied to all concentration profiles plotted in the main article as well as the SI.

The cells were first analyzed at OCV to study mass transport without applied potential. At OCV, the cumulative TEMPO and TEMPO^+^ concentrations (*i.e.*, [TEMPO] + [TEMPO^+^]) in the electrodes remained fairly constant and uniform over time and thickness in each half-cell (Fig. 2b), indicating the 20 min timeframe was too short for species diffusion through the porous separator to balance the concentration gradient.^44^ After the OCV period, alternating negative and positive voltage biases were applied, and the current output was recorded (Fig. 2a). A negative potential at the WE caused species movement due to redox reactions and the electric field,^45^ increasing species concentration in the WE and decreasing it in the CE. TEMPO was oxidized to TEMPO^+^ at the CE and migrated towards the WE, accompanied by diffusion due to the concentration gradient.^45^ Reversing the potential showed the opposite behavior, with concentration profiles converging toward the initial OCV values.

Despite the different microstructures of the paper and NIPS electrodes, their macroscopic electrochemical response is similar, both delivering a constant current output of approximately ±100 mA at an applied potential of ±0.6 V. Although the NIPS electrode has a higher electrochemically active surface area (2.9 m^2^ g^−1^)^36^ compared to the paper electrode (0.50 m^2^ g^−1^ ),^3^ the current output is similar because of the facile kinetics and low ionic conductivity of the electrolyte,^19^ as well as the porosity gradient and low permeability across the NIPS electrode thickness.^10,19,36,46^ The electrochemical performance of the cloth electrode, however, is considerably different, with a current output 2.5 times higher (average of ±240 mA over 20 min at ±0.6 V) than the paper and NIPS electrodes. The current decreases over time due to the high species conversion, which affects the SoC in the cell.^8,44^ The superior performance of the cloth electrode is attributed to its weave structure, which provides a bimodal PSD (pore diameters centered at *ca.* 20 μm and 100 μm with pores spanning 5–500 μm^3^) with large voids in the through-plane direction. This structure results in high through-plane permeability, low tortuosity (1.15–1.35), and low apparent Forchheimer coefficient (1.2 × 10^3^ m^−1^),^13^ which is beneficial in combination with low convection due to the PFF and low flow rate, and low ionic conductivity of the electrolyte.^46^

In contrast, the paper and NIPS electrodes feature differences in the concentration distributions (Fig. S2b). The NIPS electrode shows significant changes in the concentration profiles with polarity reversal, featuring a pronounced slope in the electrode thickness in the WE, from a concentration close to the tank concentration at the flow field to a higher concentration near the separator. These findings are a result of the migration of TEMPO^+^ species in response to the electric field^30^ and the diffusion of active species from the concentrated to the dilute side,^45^ influenced by the distinct electrode microstructure and amplified by low convection (PFF).^15^ The NIPS electrode contains smaller pores (∼1–5 μm) with a gradient in the electrode thickness with smaller pores facing the separator for high electrochemical activity,^10,19,37^ leading to higher tortuosity, lower apparent permeability (1.0 × 10^−11^ m^2^ for the NIPS electrode^13^*vs.* 7.4 × 10^−11^ m^2^ for the paper electrode^3^), and lower effective diffusivity compared to the paper electrode, which has an average pore size of 63 μm^3^. Consequently, we hypothesize that the NIPS electrode studied here suffers from lower convective mass transport, especially at low flow rates, causing steep concentration profiles. Conversely, the cloth electrode features more uniform profiles in the electrode thickness (Fig. S2a) due to higher convective mass transport. Moreover, the concentration changes in the CE and WE are smaller over time for all flow rates and applied potentials (Fig. S1b). The large through-plane void segments of the cloth electrode enhance diffusion and convection, resulting in smaller concentration differences across the electrode thickness and higher current output due to redox reactions occurring throughout a greater electrode volume.^46^

The concentration variations between the cloth and paper electrodes are minor compared to the significant differences in macroscopic electrochemical output. Whereas for the NIPS electrode, the macroscopic trends are similar to the paper electrode, despite differences in their concentration distribution through the cell. Since cumulative concentration profiles of [TEMPO] + [TEMPO^+^] are studied, distinguishing reactions from migration and diffusion remains challenging. Future research on alternative redox probes and computational models could help bridge this gap. Combining electrochemical performance with *operando* neutron radiography correlates the current output to concentration distributions in the cell, revealing that a bimodal PSD with large through-plane voids and high effective diffusivity is beneficial for PFF configurations with a TEMPO-based electrolyte. We then apply neutron imaging with a highly attenuating supporting ion (BF_4_^−^) and different electrolyte compositions to further investigate the transport of counter-ions and the influence of diffusion, convection, and migration on concentration profiles.

#### 3.1.2 Transport of the counter-ion

**Fig. 3:**
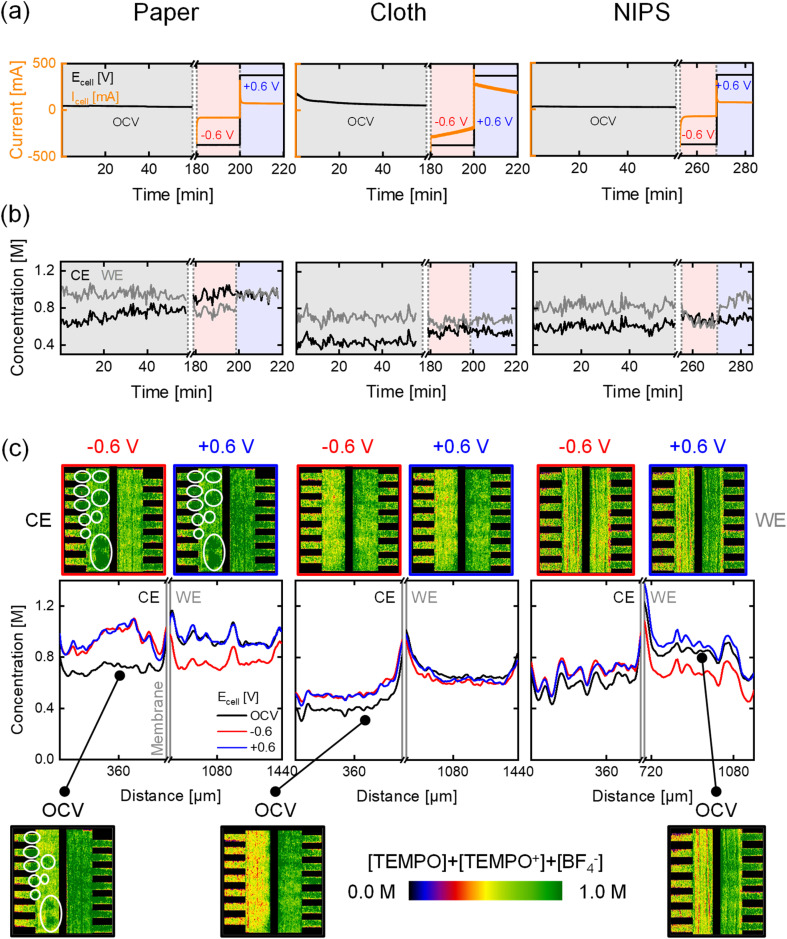
*Operando* neutron imaging of the active species transport with the attenuating BF_4_^−^ supporting ion for three electrode types: paper, cloth, and an in-house manufactured NIPS electrode, at an inlet flow rate of 5 mL min^−1^ and evaluated at OCV, −0.6 V, and +0.6 V. (a) The potential applied and current output of the electrochemical cells. (b) The averaged concentration profiles over time in the counter electrode (CE) and the working electrode (WE). (c) The cumulative active species (TEMPO and TEMPO^+^) and BF_4_^−^ supporting ion concentration profiles over the electrode thickness with the averaged snapshots of the cell after image processing, with on the counter electrode 0.5 M TEMPO and at the working electrode 0.5 M TEMPO^+^BF_4_^−^. Salt precipitation in the cell is highlighted in the snapshots.

In this section, we discuss the cumulative imaging of active species and supporting ions using the high attenuation of BF_4_^−^ (*i.e.*, [TEMPO] + [TEMPO^+^] + [BF_4_^−^]). With an electrolyte configuration of 0.5 M TEMPO at the CE and 0.5 M TEMPO^+^BF_4_^−^ at the WE (Fig. 1c), the movement of the counter-ion BF_4_^−^ can be studied. In our previous work,^30^ this configuration enabled tracking of counter-ion movement through an anion exchange membrane. However, in this work, a porous separator was used, which has no charge selectivity, permitting the transport of all species between the half-cells, enabling the study of cumulative active species and supporting ion diffusion and migration over time. The *operando* electrochemical data and concentration profiles for the three electrodes at OCV, −0.6 V, and +0.6 V for a flow rate of 5 mL min^−1^ are shown in Fig. 3. The electrochemical sequence and concentration profiles at higher flow rates and lower potentials are shown in Section S1.2, along with capacity data.

At the start of the experiments at open circuit, the OCV is not zero due to the potential difference between the half-cells caused by the initial electrolyte compositions. Over time, the OCV decreases because of electrolyte mixing between the compartments caused by species diffusion through the separator,^44^ but the time frame is not long enough to balance the concentrations in both half-cells. When a negative potential is applied, TEMPO is oxidized to TEMPO^+^ at the CE and migrates towards the WE, creating a concentration gradient. TEMPO then diffuses towards the CE through the porous separator. BF_4_^−^ migrates towards the CE to support the TEMPO oxidation reaction, resulting in an overall increase in attenuation in the CE and a decrease in the WE.^45^ When a positive potential is applied, the opposite behavior is observed, and the concentration profiles converge toward the initial OCV values.

Before analyzing the differences between the three electrodes, it is important to note that the CE in the configuration with the paper electrode cannot be analyzed quantitatively due to significant salt precipitation, affecting the neutron attenuation of the electrolyte (highlighted in Fig. 3c). Salt precipitation in the paper electrode configuration was confirmed by the absence of time-dependent changes in local concentration signals, even under polarity and flow rate reversal, indicating solid-phase formation rather than mobile dissolved species concentration. The salt precipitation resulted from not rinsing the cells between the KPF_6_ supporting salt and BF_4_^−^ counter ion experiments, as BF_4_^−^ ions precipitate when in contact with residual K^+^ ions due to the low solubility of KBF_4_ in acetonitrile.^47^ As salt precipitation adversely affects neutron attenuation and battery performance by obstructing ion transport, diminishing available surface area, and ultimately shortened battery lifespan, subsequent experiments incorporated a rinsing step using 0.5 M TEMPO solution between experiments with different electrolytes (Section 2.6). Future salt precipitation mitigation strategies could include the use of highly soluble salt pairs, implementation of intermediate electrolyte flushing cycles between different chemistries, and surface modifications of electrodes to minimize nucleation and growth of precipitates.

The electrochemical output for the paper and NIPS electrodes is again similar (∼±75 mA at ±0.6 V), supported by the capacity curves (Fig. S5) showing ∼10% conversion at 5 mL min^−1^ and −0.6 V in 20 min. The cloth electrode, however, exhibits higher current (−244 mA at −0.6 V) and capacity with a 35% conversion rate, resulting in a drop in current over time at a constant applied potential (Fig. 3a).^44^ Additionally, the current output for the cloth electrode is similar for both KPF_6_ and BF_4_^−^ experiments, whereas it is lower for the BF_4_^−^ experiment with the paper and NIPS electrodes (∼±75 mA *vs.* at ±100 mA ± 0.6 V). This difference is due to the lower ionic conductivity of the electrolytes in the BF_4_^−^ ion experiments, as no supporting salt was added. The hydraulic conductance of the paper and NIPS electrodes limit their performance, making them more sensitive to the electrolyte conductivity, while the cloth electrode's higher hydraulic conductance makes it less sensitive to these changes.^46^

Nuanced differences in the concentration distributions are observed, with the NIPS electrode showing a greater concentration slope over the electrode thickness towards the separator (Fig. S4b). These differences are however less pronounced compared to the KPF_6_ experiment due to the electrolyte solutions used. In the KPF_6_ experiment, strong diffusive mass transport from the CE to WE results in a steep concentration profile in the NIPS electrode. Conversely, in the experiment containing the BF_4_^−^, migration of the supporting anion through the porous separator is visible because of the high attenuation of this molecule. For the cloth electrode, concentration profiles in the CE and WE do not return to the OCV profiles when the electric field is reversed, due to the capacity not being fully restored. Fixing the potential and time, but not the current, prevents full capacity restoration because of the asymmetry in electrolyte solutions in the tanks. The concentration profiles in the paper (WE) and NIPS electrodes, however, converge towards the OCV profiles as ∼4 times fewer active species are converted in these electrodes. Finally, minimal differences in concentration are observed when applying a potential and changing the cell polarity with the cloth electrode, despite high species conversion (*i.e.*, an increase in the CE at negative potentials and a decrease at positive potentials). Building on these results, we further investigate the use of neutron radiography to study the impact of the flow field design on flow cell performance.

### 3.2 Impact of the flow field design

**Fig. 4:**
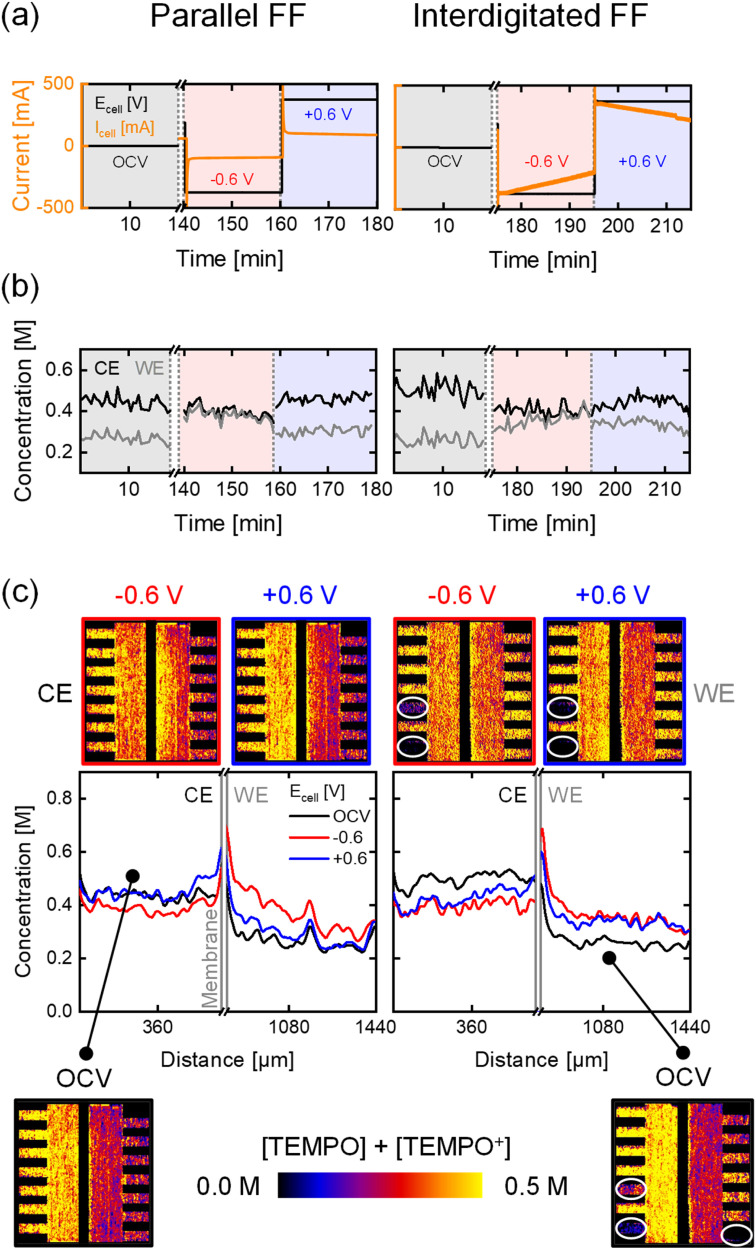
*Operando* neutron imaging of the active species transport with the low attenuating KPF_6_ supporting salt for the paper electrode and two flow field designs: parallel and interdigitated, at an inlet flow rate of 5 mL min^−1^ and evaluated at OCV, −0.6 V, and +0.6 V. (a) The potential applied and current output of the electrochemical cells. (b) The averaged concentration profiles over time in the counter electrode (CE) and the working electrode (WE). (c) The cumulative active species (TEMPO and TEMPO^+^) concentration profiles over the electrode thickness with the averaged snapshots of the cell after image processing, with on the counter electrode 0.5 M [TEMPO] + [TEMPO^+^PF_6_^−^] at 50% state-of-charge in 0.2 M KPF_6_ and at the working electrode 0.2 M [TEMPO] + [TEMPO^+^PF_6_^−^] at 50% state-of-charge in 0.2 M KPF_6_. Incomplete filling of the flow field channels is highlighted in the snapshots.

The flow field largely determines the electrolyte distribution through the electrode and the pressure drop of the system.^8,10,14^ Flow fields are commonly divided into flow-through (convection-driven) and flow-by designs (diffusion-dominated).^48^ In this work, we investigate the PFF (flow-by) and the IDFF (hybrid, predominantly flow-through), both with distinct flow distributions. The paper electrode with unimodal PSD was chosen to study the effect of convection induced by these designs on the reactor performance. In this section, imaging of active species with KPF_6_ and cumulative imaging of active species and supporting ions are discussed simultaneously. *Operando* electrochemical data and concentration profiles for the two flow fields at OCV, −0.6 V, and +0.6 V for a flow rate of 5 mL min^−1^ are shown in Fig. 4 and S8. The data at the higher flow rate and lower potentials are shown in Section S2, together with the capacity data.

Notable differences between the two flow fields are observed in the current output of the systems, which is 3 times higher with the IDFF (average of ±295 mA at ±0.6 V (KPF_6_) over 20 min, and ∼±225 mA at ±0.6 V (BF_4_^−^)) compared to the PFF (±98 mA at ±0.6 V (KPF_6_) and ∼±75 mA at ±0.6 V (BF_4_^−^)). The substantial difference is attributed to the forced convection with the IDFF, enhancing mass transport due to the higher electrolyte velocity through the electrode (eqn (2) and (3)) and increased hydraulic conductance.^15,44^ This also causes the decrease in current over time with the IDFF, as observed for the cloth electrode, supported by the capacity curves (Fig. S11).

Analyzing the concentration profiles with the IDFF (Fig. 4) reveals more uniform profiles over the electrode thickness and minimal concentration differences in the CE and WE over time for all flow rates and applied potentials, due to the convective-dominated transport compared to the PFF (Fig. S7). Similar profiles are observed with the cloth electrode and PFF, as a result of the bimodal PSD of the cloth electrode. This indicates that the ideal flow field configuration is system-dependent (*i.e.*, electrode, electrolyte). Hence, further studies should optimize RFB components considering this dependency.^9,19,43^ It must be noted that the comparison between the PFF and IDFF configurations is based on the 1D averaged concentration profiles across the electrode thickness, excluding flow field channels and unfilled regions. While 2D concentration images provide visual context, we acknowledge that the visual contrast may not fully reflect the uniformity differences.

Additionally, the neutron radiographs show improper filling of the bottom channels in the IDFF during the KPF_6_ experiments (Fig. 4c*vs.* Fig. S8c), impacting electrolyte distribution and reactive mass transport. Underutilization of the channels is associated with performance loss, which can be mitigated by proper operation (*i.e.*, channel filling) or improved manifold design. Hence, neutron imaging is a powerful tool to investigate concentration distributions and detect secondary phenomena including salt precipitation and underutilization of flow channels. We anticipate that this can particularly be helpful in assisting with the design of stacks or new reactor designs, where insights into fluid distributions and salt precipitation is difficult to obtain otherwise. This study demonstrates that neutron radiography can obtain concentration profiles over the thickness of the flow cell, providing insights into mass transport phenomena in an *operando* flow cell by studying diffusion, convection, and migration. Connecting the concentration profiles to electrochemical measurements allows us to correlate the local performance of individual cell components, such as the electrode, to cell-averaged performance data.

## 4 Conclusion

In this work, we employed *operando* neutron radiography alongside electrochemical diagnostics to study reactive mass transport in organic redox flow cells. We analyze the influence of porous electrode and flow field design on cell performance by analyzing the electrochemical performance and in-plane concentration distributions of three electrodes–paper, cloth, and an in-house manufactured non-solvent induced phase-separation electrode–and two flow field designs–parallel and interdigitated. Our findings show that distinct electrode structures have different current and concentration profiles with parallel flow fields. The non-solved induced phase-separation electrode, despite its higher electrochemical active surface area, exhibits similar macroscopic behavior to the paper electrode due to the low permeability across the electrode thickness. Neutron radiography reveals that the non-solvent induced phase-separation electrode has pronounced concentration fluctuations upon polarity reversal and a concentration gradient across the electrode thickness, attributed to its small pore size and tortuous structure. Conversely, the cloth electrode achieves higher current output due to increased capacity utilization and more uniform concentration profiles, facilitated by its bimodal pore size distribution. Moreover, interdigitated flow fields enhance through-plane electrolyte transport, resulting in greater current output at fixed potentials and flow rates. Hence, for an electrolyte with fast kinetics and low ionic conductivity, convective through-plane electrolyte transport is more crucial than a high electrochemically active surface area.

To conclude, this work highlights the importance of selecting appropriate electrode structures and flow field geometries. Neutron radiography provides valuable insights into mass transport principles, guiding advancements in reactor design and revealing secondary effects such as salt precipitation and flow field underutilization. However, it should be noted that the spatial resolution of neutron imaging in this case (∼20 μm) does not enable detection of local concentration gradients (<10 μm) within electrode pores or around electrode surfaces. As a result, localized effects such as boundary layer formation or electrolyte depletion at individual fiber interfaces are averaged into surrounding regions, potentially obscuring direct correlations with electrochemical performance. Complementary high-resolution techniques such as confocal fluorescence microscopy, electrochemical imaging, or microscale models are therefore essential to resolve local microstructural effects, diffusion boundary layers, local reaction rates, and local electrolyte velocities,^19,24^ as well as to validate neutron imaging interpretations.

## Author contributions

M.v.d.H. contributed to the conceptualization, methodology, formal analysis, investigation, data curation, visualization, image processing, writing – original draft, and writing – review and editing. R. R. J. and E. B. B. contributed to the conceptualization, methodology, formal analysis, investigation, data curation, and writing – review and editing. P. B. contributed to the methodology, formal analysis, investigation, data curation, and image processing. Finally, A. F. C. contributed to the conceptualization, methodology, formal analysis, investigation, data curation, funding, resources, writing – original draft, writing – review and editing, project administration, and supervision.

## Conflicts of interest

The authors declare no conflict of interest.

## Supplementary Material

SE-009-D5SE00844A-s001

## Data Availability

The data presented in this study can be found in the SI or can be provided by the corresponding author upon reasonable request. The following information can be found in the supplementary information: complete electrochemical and concentration profiles over time for all flow rates and potentials tested for all cell configurations, a statement on the electrode compression, concentration plots to compare the differences between the investigated cell configurations, capacity curves over time, and the main result figure for the supporting ion experiment for the flow field comparison. See DOI: https://doi.org/10.1039/d5se00844a.
