# Supplementary material for: Neutron imaging to study the influence of flow fields and porous electrodes on concentration distributions in redox flow cells
Source: Sustain Energy Fuels. 2025 Aug 5;9(19):5278–89. doi: 10.1039/d5se00844a (PMC12379122; doi:10.1039/d5se00844a)
Supplement: SE-009-D5SE00844A-s001 [file SE-009-D5SE00844A-s001.pdf]

Supplementary Information (SI) for Sustainable Energy & Fuels.
This journal is © The Royal Society of Chemistry 2025

Supporting Information

Neutron imaging to study the influence of flow fields and porous electrodes on concentration distributions in redox flow cells

Maxime van der Heijden[1,2,*], Rémy Richard Jacquemond[1], Emre Burak Boz[1,3], Pierre Boillat[4,5], Antoni Forner-Cuenca[1,3]

[1]Electrochemical Materials and Systems, Department of Chemical Engineering and Chemistry, Eindhoven University of Technology, P.O. Box 513, 5600 MB Eindhoven, The Netherlands
[2]Department of Chemical Engineering, University of Waterloo, Waterloo, ON, Canada
[3]Eindhoven Institute for Renewable Energy Systems, Eindhoven University of Technology, P.O. Box 513, 5600 MB Eindhoven, The Netherlands
[4]Electrochemistry Laboratory, Paul Scherrer Institut, Forschungsstrasse 111, CH-5232, Villigen PSI, Switzerland
[5]Laboratory for Neutron Scattering and Imaging, Paul Scherrer Institut, Forschungsstrasse 111, CH-5232, Villigen PSI, Switzerland

** Corresponding author: maxime.vanderheijden@uwaterloo.ca*

Section S1 - Influence of the electrode structure 2
S1.1. Transport of the active species 2
S1.2. Transport of the counter-ion 4
Section S2 - Impact of the flow field design 7
S2.1. Transport of the active species 7
S2.2. Transport of the counter-ion 8

Section S1 - Influence of the electrode structure

S1.1. Transport of the active species

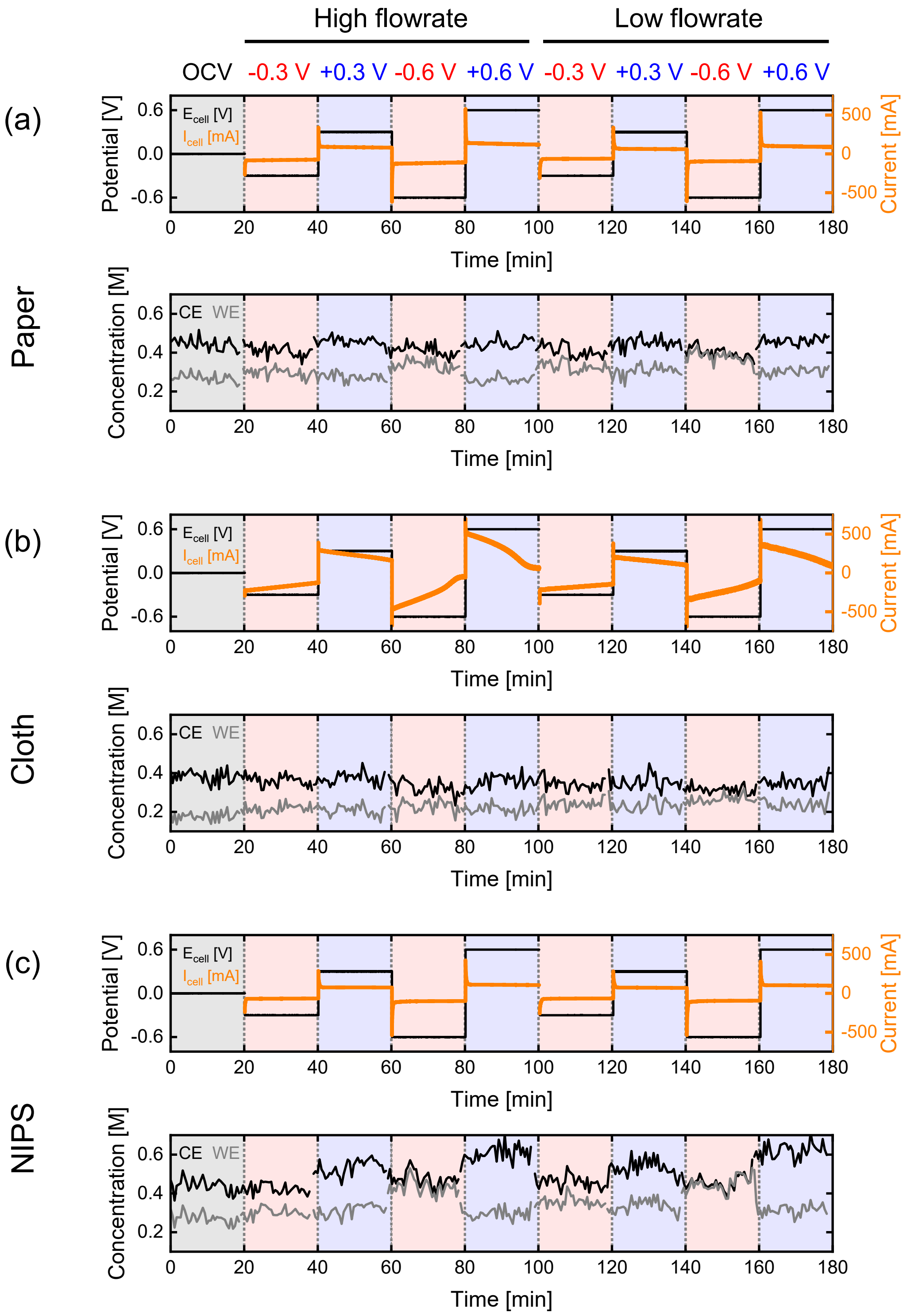

Figure S1: *Operando* neutron imaging of the active species transport with the parallel flow field and the low attenuating KPF_6 supporting salt for three electrode types: **(a)** SGL 39AA, **(b)** AvCarb Cloth, and **(c)** an in-house manufactured NIPS electrode, at an inlet flow rate of 13-15 mL min^{-1} and 5 mL min^{-1} and evaluated at OCV, -0.3 V, +0.3 V, -0.6 V, and +0.6 V. Where the potential applied and current output of the electrochemical cells are plotted over time, as well as the concentration profiles over time in the counter electrode (CE) and the working electrode (WE).

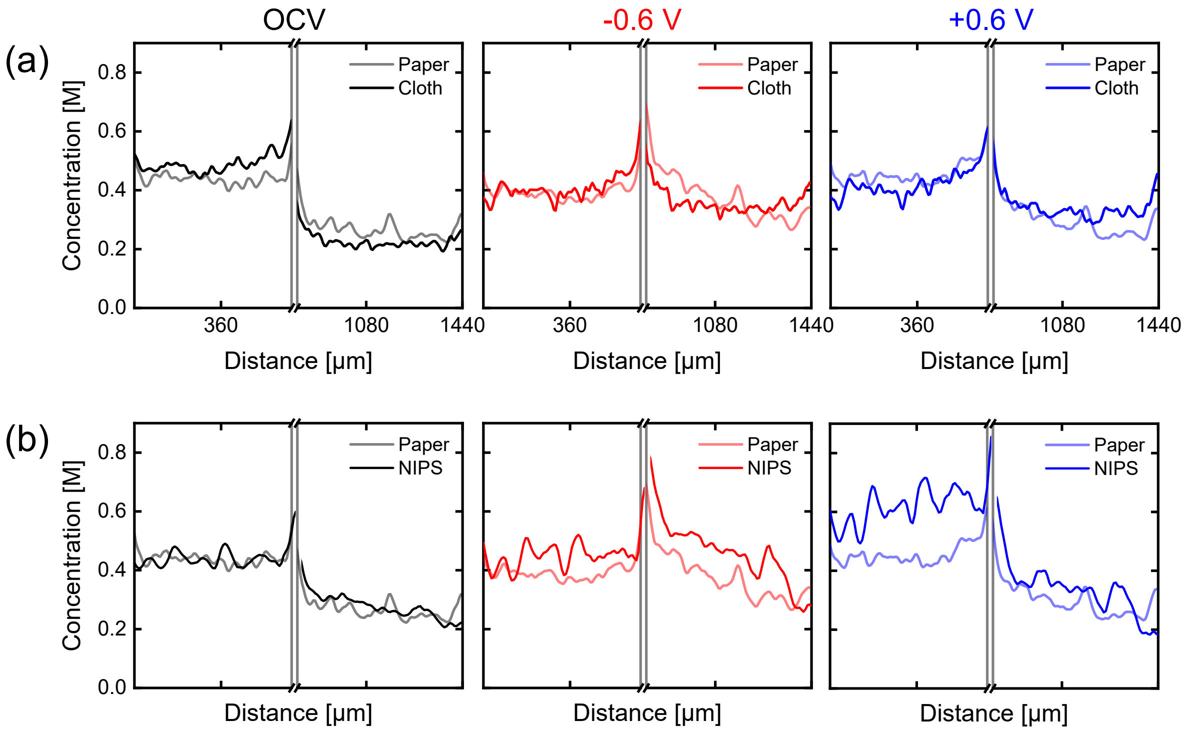

Figure S2: The cumulative active species (TEMPO and $TEMPO^+$) concentration profiles over the electrode thickness with the parallel flow field and the low attenuating KPF_6 supporting salt for three electrode types: **(a)** the SGL 39AA and the AvCarb Cloth, and **(b)** the SGL 39AA and an in-house manufactured NIPS electrode, at an inlet flow rate of 5 mL min^{-1} and evaluated at OCV, -0.6 V, and +0.6 V.

Note on Electrode Compression:

The cloth electrode (AvCarb 1186 HCB) was compressed more substantially than the other electrodes due to its initial thickness (1200 µm) in order to match the target electrode thickness (630 µm) for neutron imaging. As a result, its compressed porosity was lower (66%) compared to the paper and NIPS electrodes (both 85%). This compression may impact electrolyte distribution and contact resistance. Nonetheless, the cloth electrode exhibited enhanced through-plane transport based on the concentration distributions despite the lower compressed porosity. Moreover, although compression increases surface area per unit thickness, the electrochemically active surface area of the cloth electrode remained substantially lower than that of the other electrodes, even after compression. Finally, we chose to compare electrodes at similar electrode thickness instead of compression, as for the kinetically facile electrolyte with low ionic conductivity, the ionic resistance as a result of the electrode thickness significantly impacts the overpotential in the cell. These observations suggest that compression alone does not account for improved performance. Still, readers should consider porosity and compression discrepancies when interpreting cross-electrode comparisons.

S1.2. Transport of the counter-ion

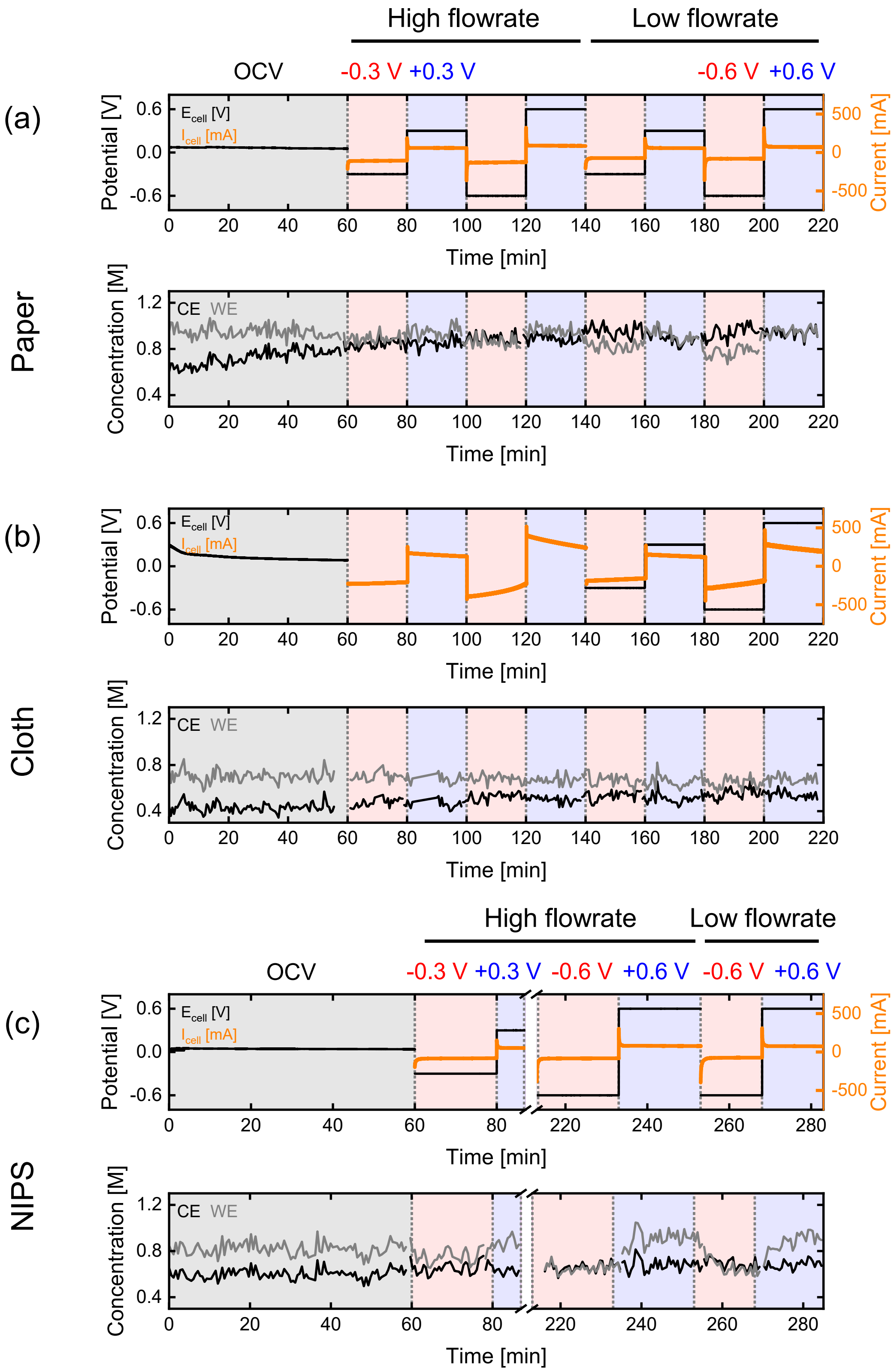

Figure S3: *Operando* neutron imaging of the active species transport with the parallel flow field and the attenuating BF4$^-$ supporting ion for three electrode types: **(a)** SGL 39AA, **(b)** AvCarb Cloth, and **(c)** an in-house manufactured NIPS electrode, at an inlet flow rate of 13-15 mL min^{-1} and 5 mL min^{-1} and evaluated at OCV, -0.3 V, +0.3 V, -0.6 V, and +0.6 V. Where the potential applied and current output of the electrochemical cells are plotted over time, as well as the concentration profiles over time in the counter electrode (CE) and the working electrode (WE).

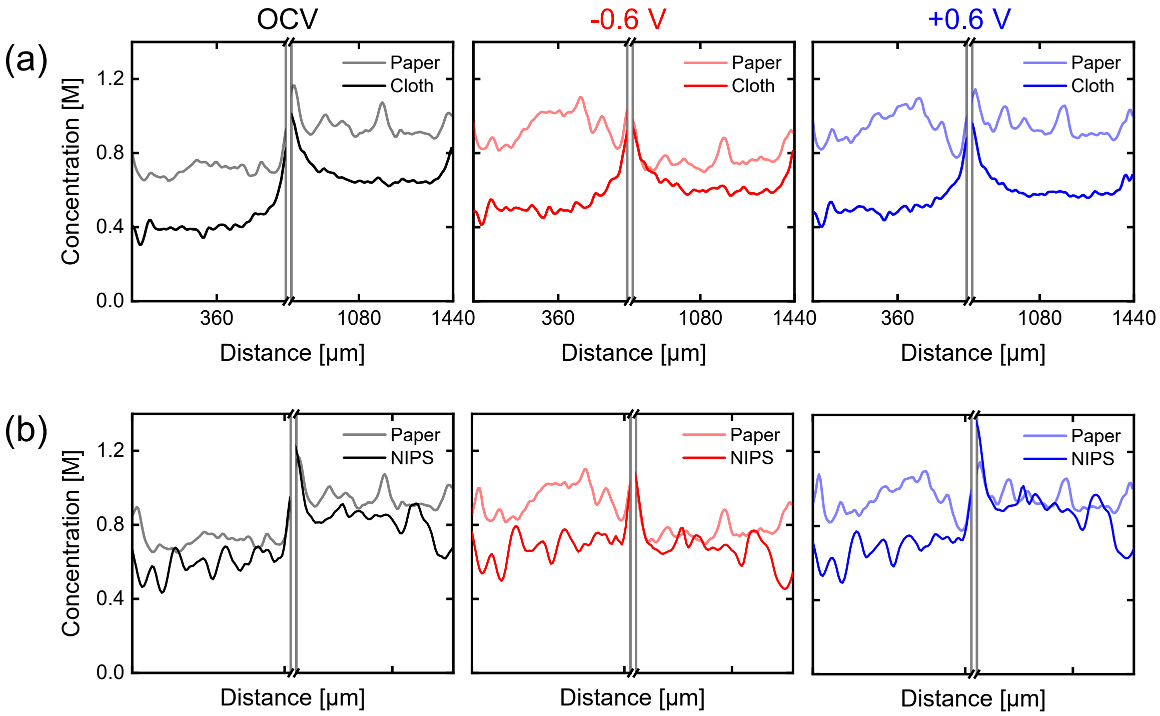

Figure S4: The cumulative active species (TEMPO and $TEMPO^{+}$) and BF_4^- supporting ion concentration profiles over the electrode thickness for three electrode types: **(a)** the SGL 39AA and the AvCarb Cloth, and **(b)** the SGL 39AA and an in-house manufactured NIPS electrode, with the parallel flow field, at an inlet flow rate of 5 mL min^{-1}, and evaluated at OCV, -0.6 V, and +0.6 V.

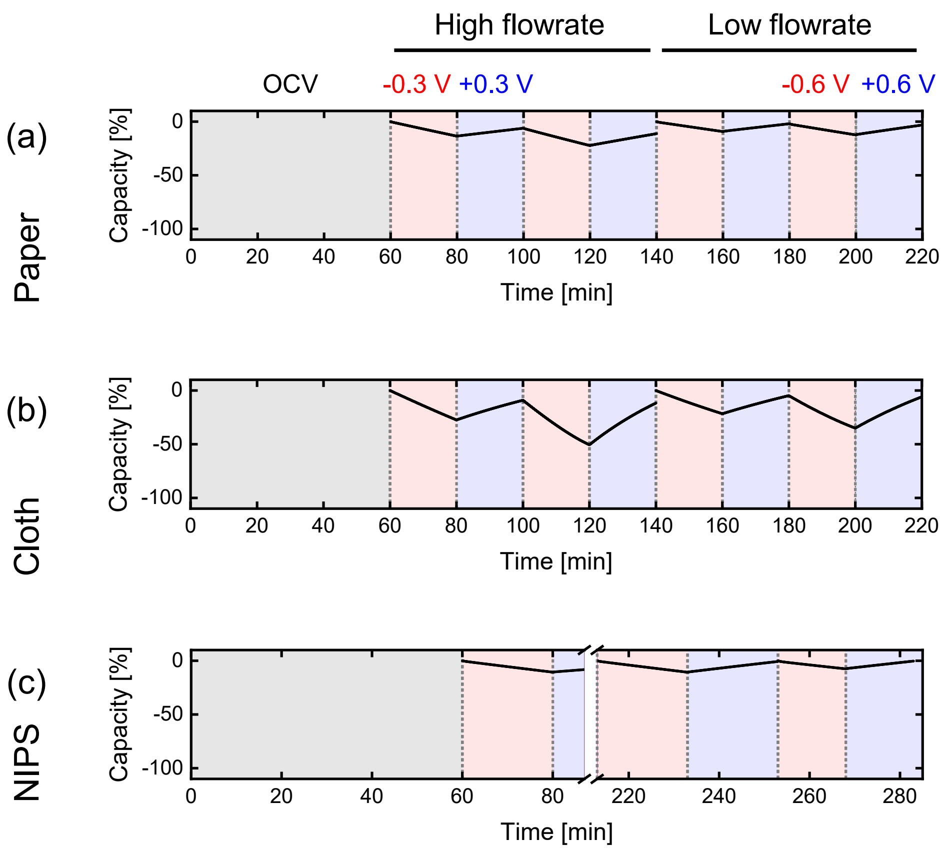

Figure S5: *Operando* neutron imaging of the active species transport with the parallel flow field and the attenuating $BF4^-$ supporting ion for three electrode types: **(a)** SGL 39AA, **(b)** AvCarb Cloth, and **(c)** an in-house manufactured NIPS electrode, at an inlet flow rate of 13-15 mL min^{-1} and 5 mL min^{-1} and evaluated at OCV, -0.3 V, +0.3 V, -0.6 V, and +0.6 V. Where the capacity of the electrochemical cells is plotted over time.

Section S2 - Impact of the flow field design

S2.1. Transport of the active species

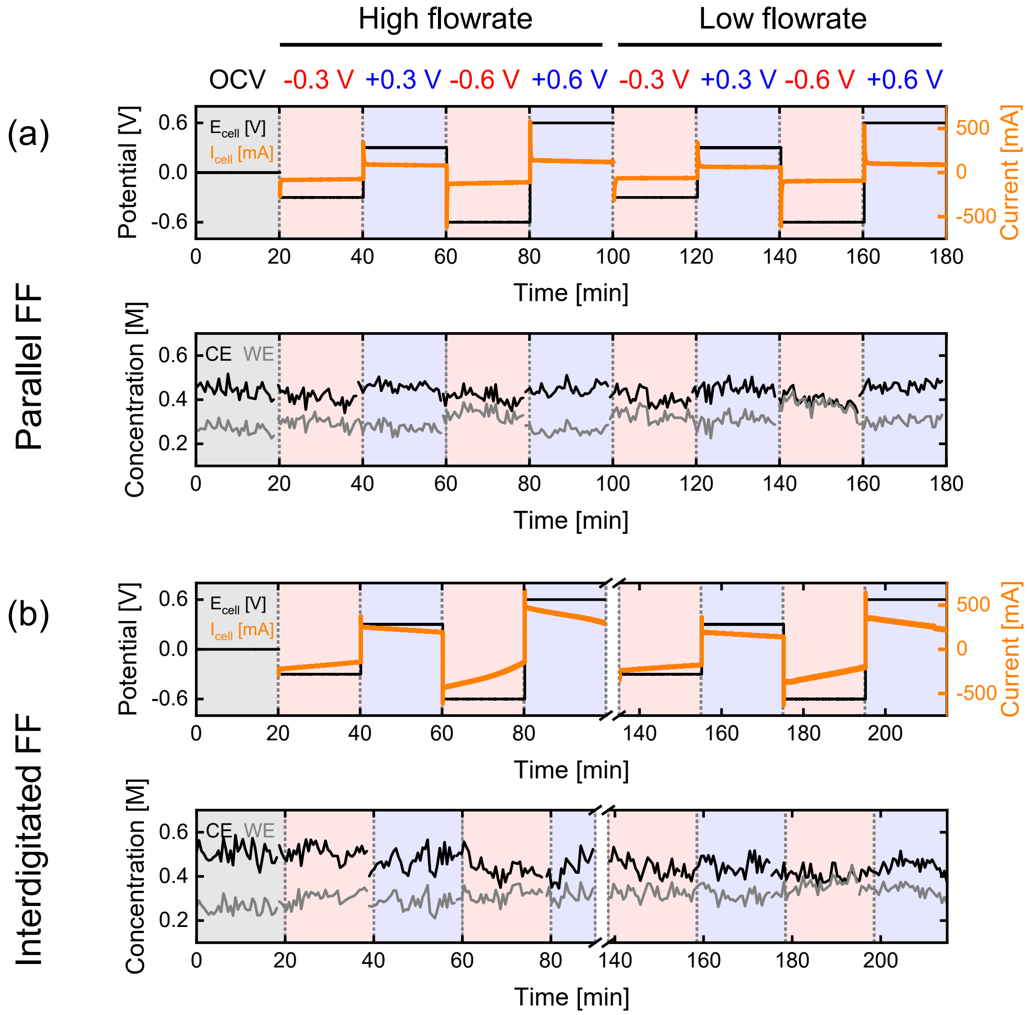

Figure S6: *Operando* neutron imaging of the active species transport with the paper electrode and the low attenuating KPF_6 supporting salt for two flow field designs: **(a)** parallel, and **(b)** interdigitated, at an inlet flow rate of 15 mL min^{-1} and 5 mL min^{-1} and evaluated at OCV, -0.3 V, +0.3 V, -0.6 V, and +0.6 V. Where the potential applied and current output of the electrochemical cells are plotted over time, as well as the concentration profiles over time in the counter electrode (CE) and the working electrode (WE).

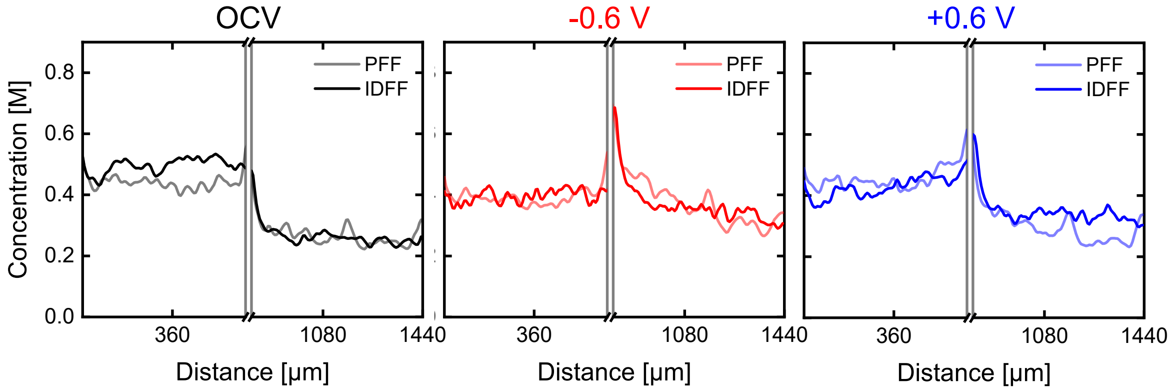

Figure S7: The cumulative active species (TEMPO and $TEMPO^+$) concentration profiles over the electrode thickness with the paper electrode and the low attenuating KPF_6 supporting salt for the parallel and interdigitated flow fields, at an inlet flow rate of 5 mL min^{-1} and evaluated at OCV, -0.6 V, and +0.6 V.

S2.2. Transport of the counter-ion

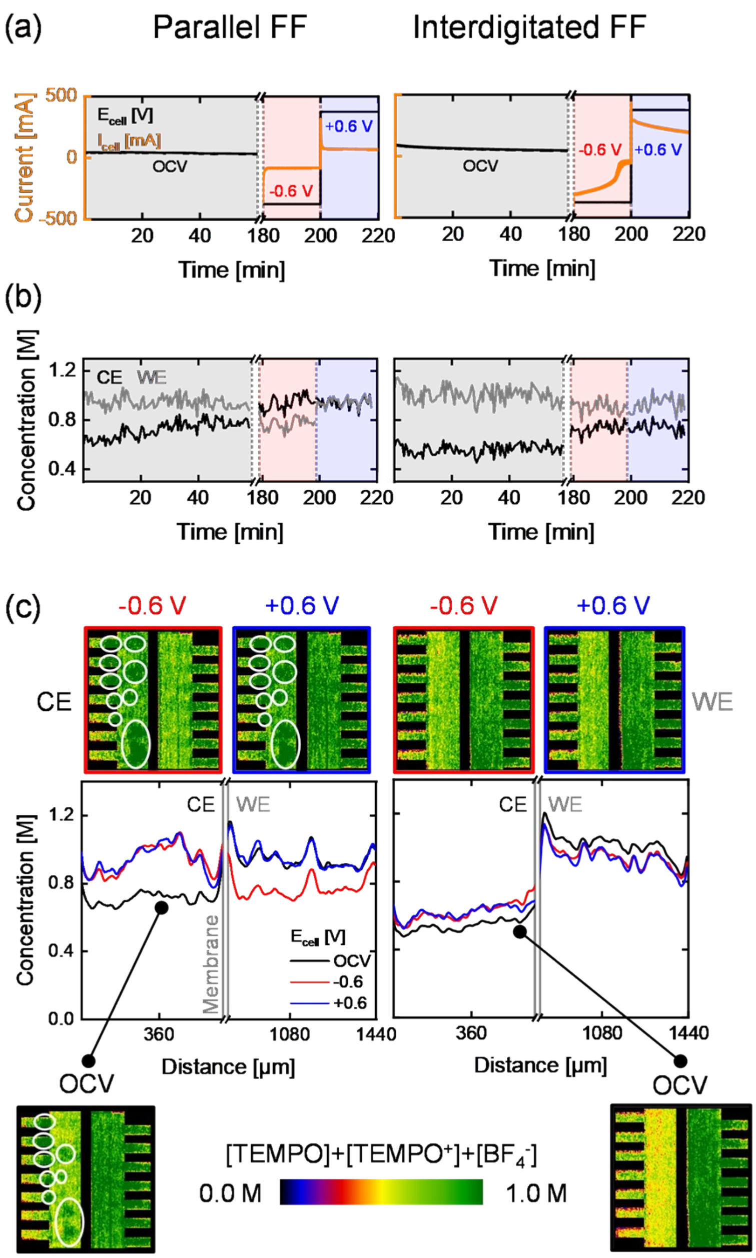

Figure S8: *Operando* neutron imaging of the active species transport with the paper electrode and the attenuating BF4^{-} supporting ion for two flow field designs: parallel and interdigitated, at an inlet flow rate of 5 mL min^{-1} and evaluated at OCV, -0.6 V, and +0.6 V. **(a)** The potential applied and current output of the electrochemical cells. **(b)** The averaged concentration profiles over time in the counter electrode (CE) and the working electrode (WE). **(c)** The cumulative active species (TEMPO and TEMPO^{+}) and BF_4^{-} supporting ion concentration profiles over the electrode thickness with the averaged snapshots of the cell after image processing, with on the counter electrode 0.5 M TEMPO and at the working electrode 0.5 M TEMPO^{+}BF_4^{-}. Salt precipitation in the cell is highlighted in the snapshots.

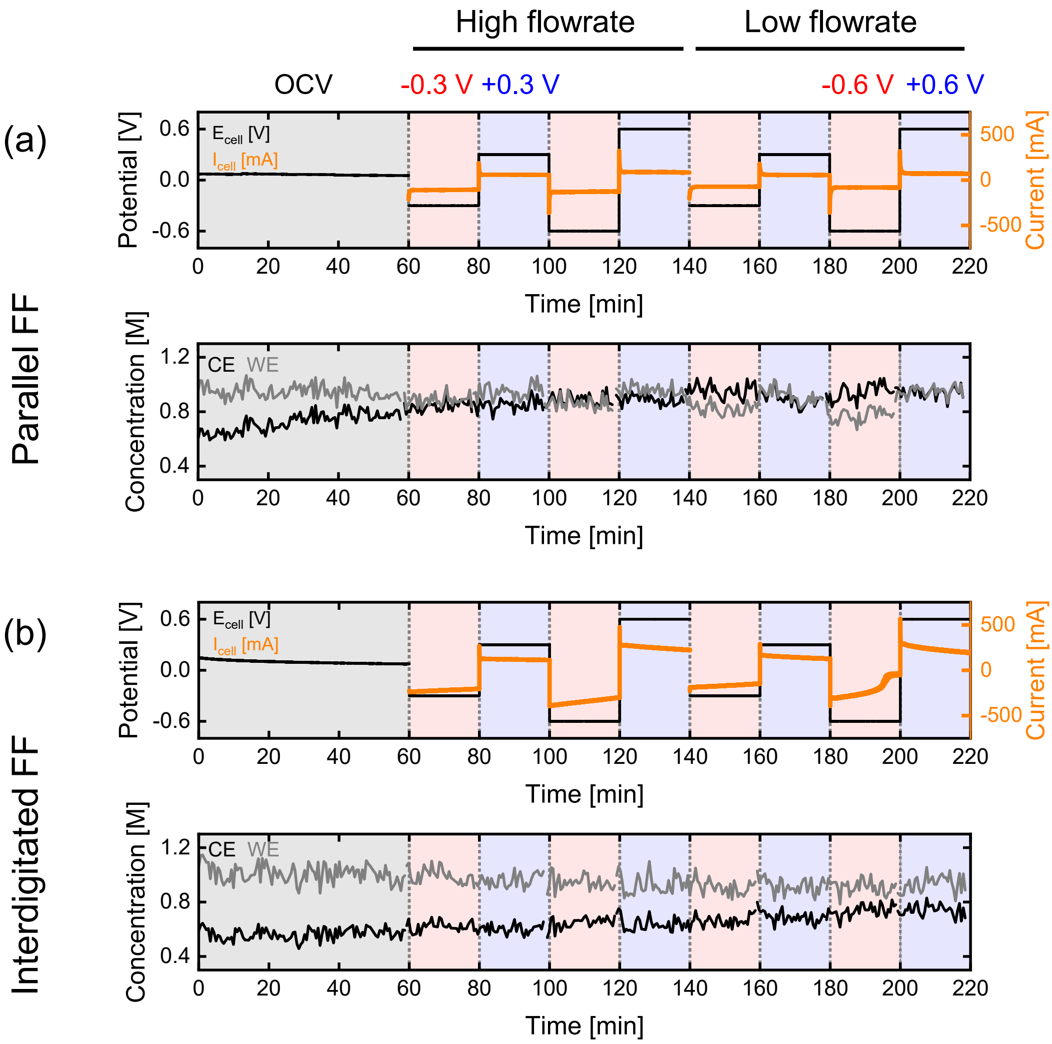

Figure S9: *Operando* neutron imaging of the active species transport with the paper electrode and the attenuating BF4$^-$ supporting ion for two flow field designs: **(a)** parallel, and **(b)** interdigitated, at an inlet flow rate of 15 mL min^{-1} and 5 mL min^{-1} and evaluated at OCV, -0.3 V, +0.3 V, -0.6 V, and +0.6 V. Where the potential applied and current output of the electrochemical cells are plotted over time, as well as the concentration profiles over time in the counter electrode (CE) and the working electrode (WE).

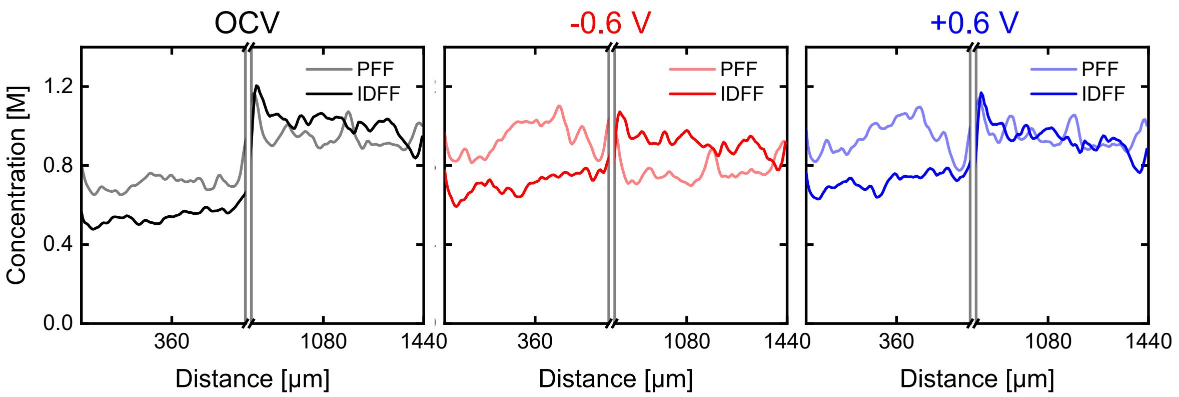

Figure S10: The cumulative active species (TEMPO and TEMPO$^+$) and BF_4^- supporting ion concentration profiles over the electrode thickness with the paper electrode for the parallel and

interdigitated flow fields, at an inlet flow rate of 5 mL min^{-1}, and evaluated at OCV, -0.6 V, and +0.6 V.

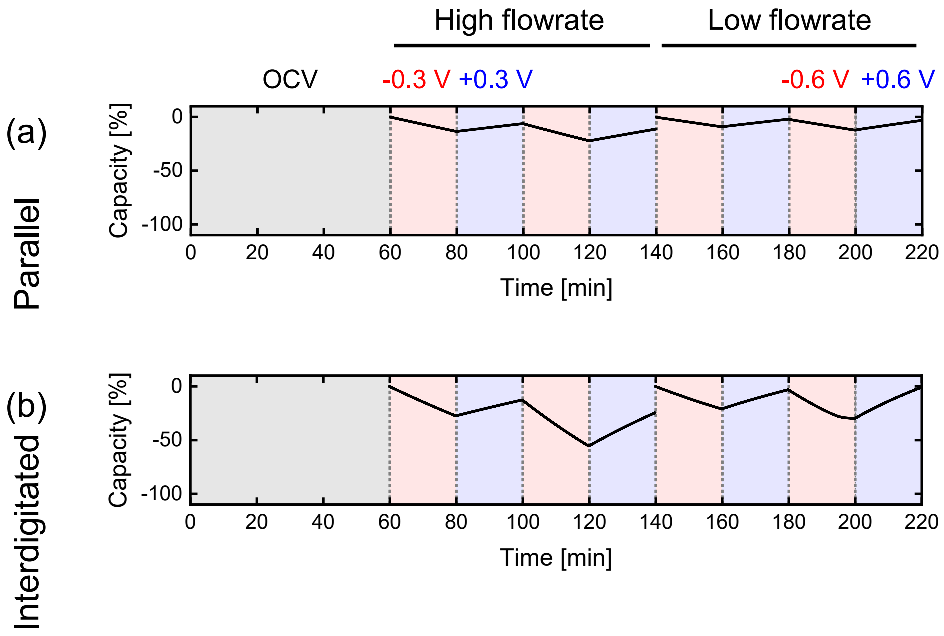

Figure S11: *Operando* neutron imaging of the active species transport with the paper electrode and the attenuating $BF4^-$ supporting ion for two flow field designs: **(a)** parallel, and **(b)** interdigitated, at an inlet flow rate of 15 mL min^{-1} and 5 mL min^{-1} and evaluated at OCV, -0.3 V, +0.3 V, -0.6 V, and +0.6 V. Where the capacity of the electrochemical cells is plotted over time.
